# Editorial: Cutaneous oncology and skin cancer genomics

**DOI:** 10.3389/fmed.2024.1445026

**Published:** 2024-08-05

**Authors:** Bahar Dasgeb

**Affiliations:** Rutgers Cancer Institute of New Jersey, Rutgers, The State University of New Jersey, New Brunswick, NJ, United States

**Keywords:** cutaneous oncology, skin genomics, complex skin malignancies, complex skin tumors, adverse events, genomics of skin malignancies

The past 30 years has witnessed an incremental incidence and prevalence of skin malignancies—the most common malignancy in humans globally—with a recent predilection for younger age and a measurable social and economic impact on healthcare systems. This has happened in light of measures taken for sun protection and early detection during this period. Accordingly, further studies are warranted to better understand the additional risk factors underlying the surge in skin malignancies.

In this Research Topic, valuable research and observations of contributing authors shed light on a wide range of aspects of cutaneous malignancies. Included, are two comprehensive review articles on cutaneous angiosarcoma (Guan et al.) and melanoma (Waseh and Lee), both of which do commendably bring the readers up to date with the current knowledge, the cutting-edge developments, and the ongoing progress in the horizon on both subjects. The presented body of work has also made an effort to emphasize the basic importance of clinical risk stratification for prevention and early detection (Banner et al.; Park et al.) along with the emerging role of technology and AI in dermatology (Schreidah et al.).

Beyond prevention and early detection, we have also shed a light on Complex Cutaneous Malignancies (CCM) comprised of numerous, locally advanced, repeatedly recurring, resistant to treatment, which can present in less-sun-exposed or non-sun-exposed skin. These complex skin tumors may present an opportunity for investigating the impact of inherited or acquired oncogenic genes in relation to other risk factors, including sun (Gandarillas, Tang et al.). The contributing prospective of oncogenic genes in increasing incidence of skin malignancies in light of sun protective practices of the past few decades is an important area of investigation, which is reflected in the title of presented Research Topic; “*Cutaneous oncology and skin cancer genomics*” (Warbasse et al.; King et al.). Moreover, understanding of individuals' genomics, including but not limited to HLA system has presented a potential in risk stratification of patients and management of the treatment associated adverse events that warrants attention (Bhatti et al.; Gandarillas, Newland et al.).

I would like to conclude this note in memory of Dr. Jouni Uitto, a co-editor of this Research Topic, whose friendship and mentorship shaped my career. His contagious laugh even in testing times was a gift to all of us around him. I cherish his memories, especially the most impactful moments, when I learned the most from him about life and science, such is this summer afternoon ice cream moment (Figure 1).

**Figure 1:**
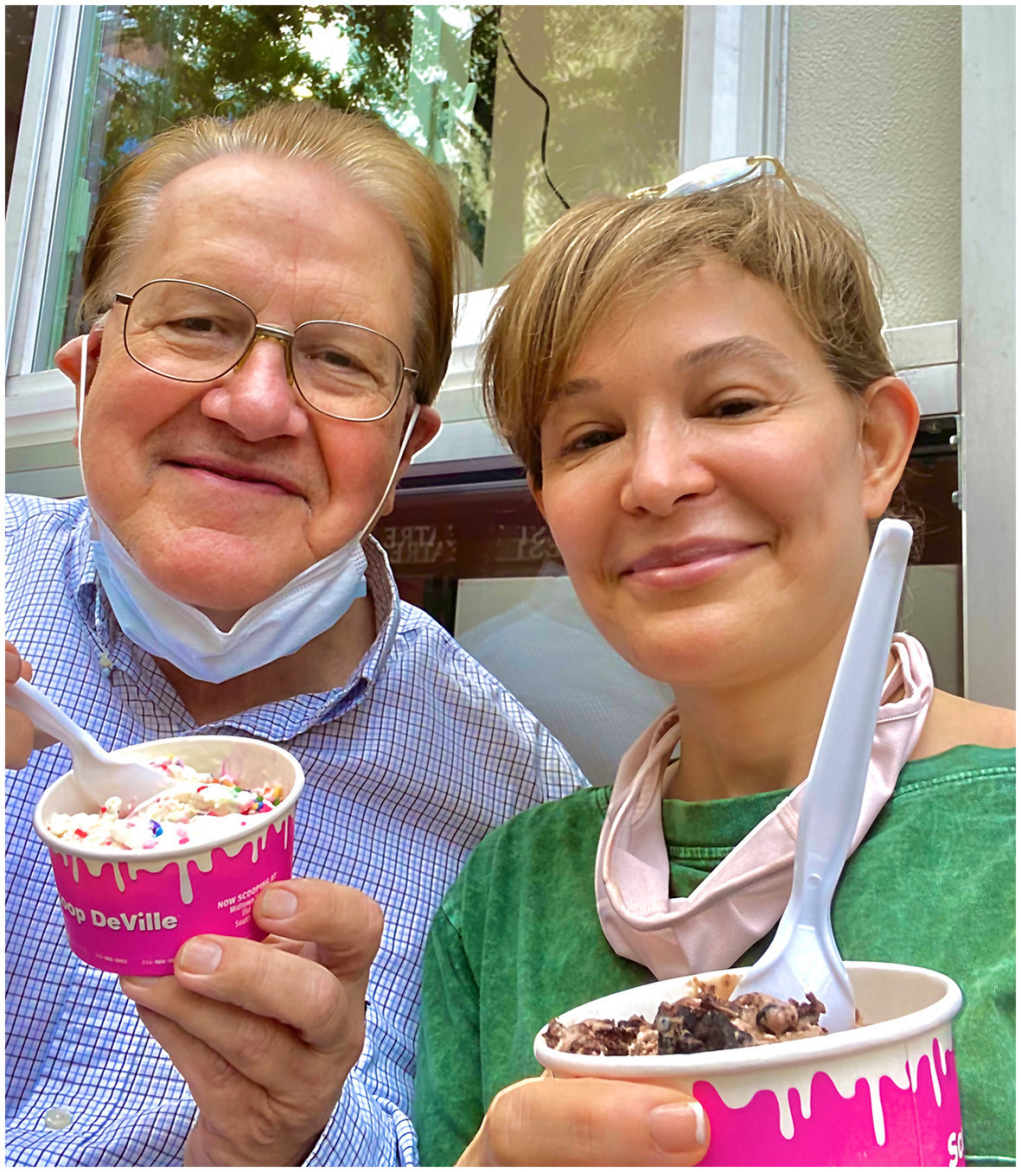
Summer afternoon ice cream moment, Dr. Jouni Uitto **(Left)** and author BD **(Right)**.

On the same note, I would like to express my gratitude for the opportunity of collecting this body of work through which valuable friendships and collaboration was built not only with the contributing authors but also with the outstanding staff of Frontiers in Medicine, especially Josie Wyatt whose professional administrative support was priceless during this process.

## Ethics statement

Written informed consent was obtained from the individual(s) for the publication of any identifiable images or data included in this article.

## Author contributions

BD: Conceptualization, Data curation, Formal analysis, Investigation, Methodology, Project administration, Resources, Supervision, Validation, Visualization, Writing – original draft, Writing – review & editing.

